# Endoscopic ultrasound-guided salvage technique for pancreatic duct injury during endoscopic papillectomy

**DOI:** 10.1055/a-2418-3257

**Published:** 2024-10-08

**Authors:** Haruo Miwa, Kazuki Endo, Ritsuko Oishi, Yugo Ishino, Shotaro Tsunoda, Yuichi Suzuki, Shin Maeda

**Affiliations:** 1 26437 Gastroenterological Center, Yokohama City University Medical Center, Yokohama, Japan; 2 26438 Gastroenterology, Yokohama City University School of Medicine Graduate School of Medicine, Yokohama, Japan

Pancreatic stent placement is suggested after endoscopic papillectomy 1 2 ; however, there is a risk of pancreatic duct injury during guidewire manipulation. Endoscopic ultrasound-guided pancreatic duct drainage (EUS-PDD) can be an alternative technique after failed endoscopic retrograde pancreatography 3 4 .

A 53-year-old woman was referred to our hospital with an ampullary tumor. We performed endoscopic papillectomy because the lesion was localized to the papilla. En bloc resection of the papilla of Vater was performed using an electrical snare. The anal side of the wound was sutured using hemoclips. Pancreatic duct cannulation was attempted for placement of a pancreatic stent; however, it failed because of guidewire penetration into the retroperitoneal cavity ( Fig. 1 ).

**Fig. 1:**
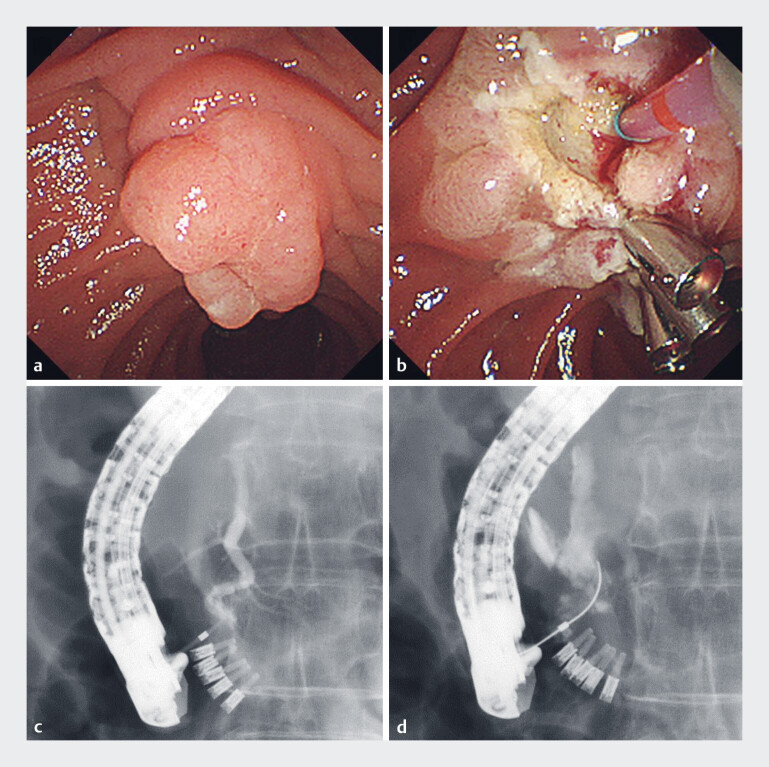
Images during endoscopic papillectomy showing: **a, b** on endoscopic view: **a** an ampullary tumor that was localized to the papilla; **b** pancreatic duct cannulation being attempted after prophylactic clipping had been carried out; **c, d** on fluoroscopic view; **c** significant bends within the main pancreatic duct; **d** pancreatic duct injury caused by guidewire penetration into the retroperitoneal cavity.

EUS-PDD was planned as a salvage technique to place pancreatic duct stents both upstream and downstream ( Fig. 2 ). A nondilated main pancreatic duct was punctured in the pancreatic body using a 22-gauge needle. After the injection of contrast, a 0.018-inch guidewire was inserted into the pancreatic duct. An ultratapered catheter (MTW Endoskopie Manufaktur, Wesel, Germany) was inserted into the pancreatic duct, and the guidewire was exchanged for a 0.025-inch one. After the guidewire had been advanced into the duodenum, a double-lumen catheter was inserted to deploy an additional guidewire. A plastic stent (7-Fr, 12-cm REGULUS biliary tube stent system; Japan Lifeline Co. Ltd., Tokyo, Japan) was placed via the transgastric route following mechanical dilation. Subsequently, a duodenoscope was inserted and pancreatic duct cannulation was successfully performed along the antegrade guidewire. Finally, a plastic stent (7-Fr, 3-cm Through & Pass; Gadelius Medical, Tokyo, Japan) was placed through the papilla into the pancreatic duct ( Fig. 3 ; Video 1 ). The patient was discharged on the 7th day after the endoscopic papillectomy, without any symptoms.

**Fig. 2:**
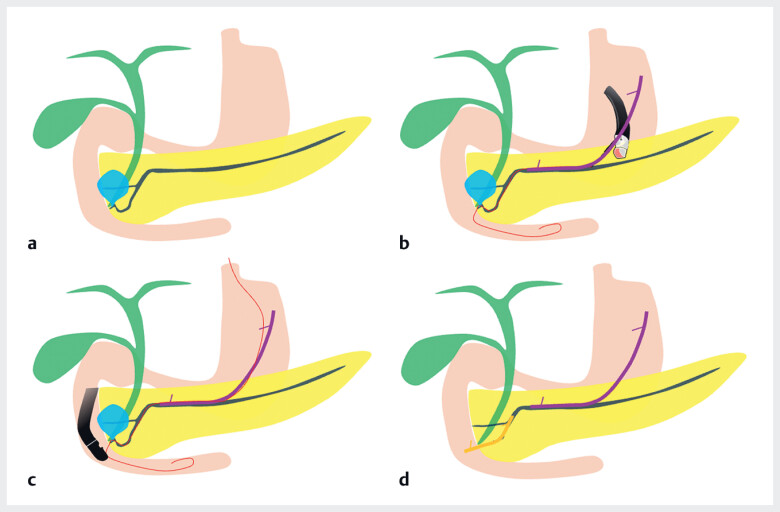
Schemas of the endoscopic ultrasound (EUS)-guided salvage technique for pancreatic duct injury showing: **a** a pancreatic duct injury; **b** EUS-guided pancreatogastrostomy; **c** pancreatic cannulation along the antegrade wire; **d** transpapillary pancreatic stent placement.

**Fig. 3:**
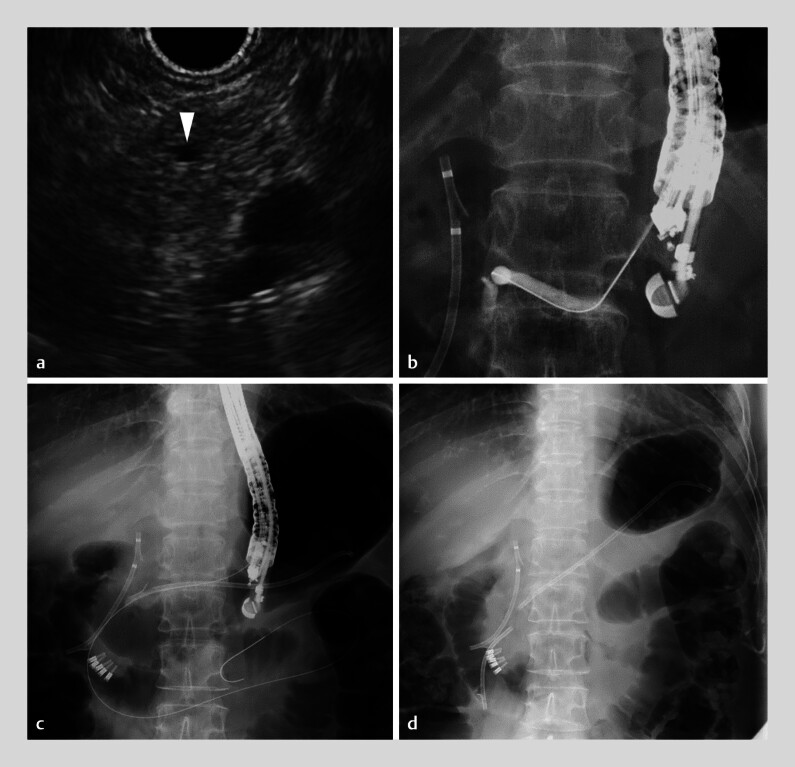
Images during the endoscopic ultrasound (EUS)-guided salvage procedure showing: **a** a 1.5-mm main pancreatic duct (arrowhead) on the EUS image; **b** puncture of the main pancreatic duct using a 22-gauge needle; **c** EUS-guided pancreatogastrostomy being performed while the antegrade guidewire remains; **d** the successfully placed transpapillary pancreatic stent.

Endoscopic ultrasound-guided pancreatic duct drainage was performed as a salvage technique following pancreatic duct injury during endoscopic papillectomy.Video 1

To the best of our knowledge, this is the first report of an EUS-guided salvage technique being used for pancreatic duct injury during endoscopic papillectomy.

Endoscopy_UCTN_Code_TTT_1AS_2AI

## Notes

Endoscopy E-Videos https://eref.thieme.de/e-videos
*E-Videos* is an open access online section of the journal *Endoscopy* , reporting on interesting cases and new techniques in gastroenterological endoscopy. All papers include a high-quality video and are published with a Creative Commons CC-BY license. Endoscopy E-Videos qualify for HINARI discounts and waivers and eligibility is automatically checked during the submission process. We grant 100% waivers to articles whose corresponding authors are based in Group A countries and 50% waivers to those who are based in Group B countries as classified by Research4Life (see: https://www.research4life.org/access/eligibility/ ). This section has its own submission website at https://mc.manuscriptcentral.com/e-videos .
